# Kairomonal communication in mice is concentration-dependent with a proportional discrimination threshold

**DOI:** 10.12688/f1000research.2-195.v2

**Published:** 2013-12-09

**Authors:** Anand Vasudevan, Ajai Vyas

**Affiliations:** 1 School of Biological Sciences, Nanyang Technological University, 637551, Singapore

## Abstract

Odors of predators are often co-opted by prey species to serve as warning signals. Perceptual properties of such kairomonal communication are under studied despite their common use in many mammals. We demonstrate that the kairomonal response in mice to rat odors varies monotonically with the volume of rat odor. Moreover, the ability of mice to differentiate between two strengths of rat odors is dependent on the ratio of the two concentrations. These results show that mice can compare kairomonal strength over a large range of values, and that kairomonal communication follows Weber’s law.

## Introduction

Foraging animals continually face a conflict between 1) the need to seek opportunities such as food and mating partners; and, 2) the need to avoid exposure to predators. In response to predation pressure, many prey species have co-opted predator odors as kairomones; chemicals emitted by one species, usually for inter-species communication, but intercepted by other species resulting in benefit for the receiver and detriment of the emitter. In this role, predator odors such as urine, fecal material or body odors initiate a rapid avoidance response in prey, thus reducing the probability of successful predation ^1, 2^. Such avoidance of predator cues needs to be ‘traded-off’ against foraging opportunities. In view of this, it can be speculated that a kairomonal responses may not be an absolute all-or-nothing phenomenon. Rather avoidance is expected to be relative to the intensity of the predator cue. Implicit in this speculation is the idea that animals can quantitatively perceive differences in kairomonal strength.

A wide variety of animals can make quantitative estimates of percepts such as time, foraging opportunities, efforts and rewards ^3– 7^. These quantitative estimates are often derived using a comparative representational system that is dependent on the ratios between opposing quantities ^3– 7^. The ability to make quantitative estimates is important because it allows calibration of behavioral responses to incipient environmental opportunities and challenges. In accordance with the comparative nature of such perceptual systems, it can be predicted that greater quantities of kairomones evoke greater response i.e. that the response is dose-dependent. More importantly, sensitivity to changes in the magnitude of a stimulus decreases when stimulus magnitude increases. In other words, the discrimination threshold (i.e. the ‘just-noticeable difference’ between two stimuli of different intensities) is smaller when both stimuli are weak compared to when both stimuli are strong. This formulation is often termed Weber’s law ^8^, and is a fundamental property of many percepts.

Kairomonal communication has been widely studied in insects ^9– 12^. Additionally, the neurobiology and physiology of rodent kairomones has attracted significant scientific interest in the recent past ^1, 13^. Yet, the perceptual properties of kairomonal communication in mammals have so far been under studied, including, the dose-responsivity of kairomonal communication ^14^ and the relationship of discrimination threshold to stimulus magnitude.

House mice ( *Mus musculus*) are predated by rats ( *Rattus norvegicus*) ^15– 17^ and accordingly, the mice express innate avoidance to rat odors ^18, 19^. In this report, we investigate the dose-responsivity and discrimination threshold of kairomonal communication in mice.

## Materials and methods

### Animals

The Nanyang Technological University (IACUC number: ARF SBS/NIE-A-0106AZ) institutional animal care and use committee reviewed and approved all procedures. Fifteen male Balb/c mice (7–8 weeks old, housed five per cage (369 x 156 x 132 mm; 1145T, Tecniplast, UK)) were obtained from the vivarium of the National University of Singapore. Eight male Wistar rats (48 days old, housed two per cage (425 x 266 x 185 mm; 1291H, Tecniplast, UK)) were obtained from the same vivarium and used as a kairomonal source. Standard corn cob cage bedding was changed twice a week. Animals were maintained on a 12 hours light-dark cycle, with temperature and relative humidity ranging between 20–25 degree celsius and 70–80%, respectively. Experiments were conducted during the light phase. Food and water was available *ad libitum*. The diet consisted of standard laboratory chow (PicoLab Rodent Diet 20, 5053) with 20% protein content.

### Kairomone collection

Rat urine was collected using metabolic cages (Harvard Apparatus). A single pool of rat urine was used for all subsequent experimentations. Rat urine contains volatile compounds and major urinary proteins (MUPs). The urine was treated with menadione (M5625 Sigma-Aldrich, Singapore) to competitively displace volatile compounds bound to the MUPs, followed by centrifugation (Millipore, 3000 g for 5 minutes) through a size-exclusion column (>3 kDa). Only the high molecular-weight fraction containing MUPs and devoid of volatiles was used, in accordance with the prior demonstration that rat MUPs serve as kairomones to mice ^13^.

### Dose-responsivity to kairomones

The response of mice (n = 10) to increasing doses of MUP fraction of rat urine ((henceforth referred to as rat urine) was studied (trial duration = 600s). Avoidance was quantified by comparing time spent by mice in two opposing bisects of an arena (76 × 9 cm; 15 cm high). The bisects were defined by a virtual division of the arena in two equal halves (38 × 9 cm), with exploration in either half being considered as time spent near the stimulus presented in that bisect. The stimuli were presented at the terminal end of the bisects. Data on time spent in each bisect was collected by automated behavioral tracking software (ANY-maze, version 4.3, Stoelting). Opposing arms contained either rat urine or phosphate-buffered saline. The amount of rat urine was systematically varied from 1X to 16X (3.125, 6.25, 12.5, 25 and 50 µl). X was arbitrarily defined as 3.125 µl of rat urine. The different stimuli were made by a twofold serial dilution to ensure that a constant volume was presented (50 µl) while the concentration varied. The stimuli was dotted (5 drops of 10 µl) on filter paper and positioned at the terminal ends of the two bisect. The animals had direct access to the stimuli. The same set of mice was used in successive testing for all doses (starting from lower to higher doses) with 24 hours elapsing between two successive trials).

### Discrimination threshold

Both arms of the arena contained rat urine in this condition. The amount of rat urine in one arm was varied in five discrete doses (6.25–25 μl), equidistant on a Log _2_ scale. The stimuli was prepared in the similar way as before ensuring an equal volume was used, with only the concentration being varied. The opposing arm contained volume that was greater by ratio of either 1.2 or 1.3. The percentage of time that mice (n = 15, the same mice that were used in the previous experiment) spent in the arm with the greater volume of urine, was quantified. The same set of mice were used in successive testing for all doses (starting from lower to higher doses), with two successive trials (24 hours apart).

### Statistics

All statistical tests were conducted using IBM SPSS software (version 20) One-way analysis of variance followed by ‘Fisher’s least significant difference’ (LSD) post-hoc test was used to analyze increasing doses of kairomones on mouse behaviour. A two-way analysis of variance was carried out to determine main effect and/or interaction of kairomone dose and its corresponding ratios.

## Results

### Dose responsivity of kairomonal communication

**Figure 1.**
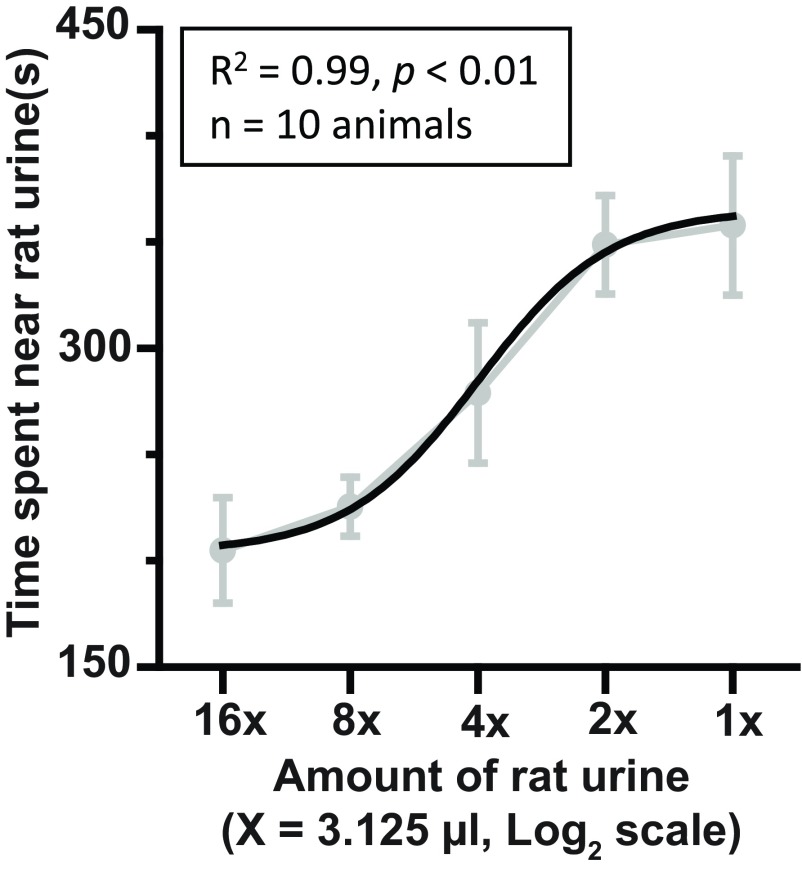
Kairomonal communication in mice is dose-dependent (mean±SEM). Aversion of mice to increasing doses of rat urine was quantified by comparing the time spent in two opposing arms of an arena, with one arm containing incremental doses of rat urine and the other containing buffered saline (trial duration = 600 s, n = 10 mice). The graph depicts average time spent in rat urine arm. The gray line depicts sigmoidal fit. Abscissa depicts dose of rat urine employed (log _2_ scale; x arbitrarily set as 3.125 µl of rat urine).

One-way analysis of variance (ANOVA) revealed that the amount of time spent near rat urine decreased with an incremental increase in the amount of rat urine ( Figure 1; n = 10; F _(4,45)_ = 6.9; *p* = 0.0002). Animals spent significantly more time near the weakest odor, compared to the strongest (LSD, Fisher's least significant difference, *p* = 0.0002). The stimulus-response curve exhibited a robust fit to sigmoidal curve ( Figure 1; R ^2^ > 0.99; *p* < 0.01), showing a monotonic linear response between concentrations 2X to 8X.

In order to rule out carry-over effects during repeated trials, we tested a separate set of five mice repeatedly at a singular dose (4X) over five days. This set of mice exhibited a comparable aversion to rat urine across all trials, showing a lack of habituation, sensitization or conditioning during repeated testing (one-way ANOVA; F _(4,20)_ = 0.314, *p* > 0.8).

### Proportional discrimination threshold

**Figure 2.**
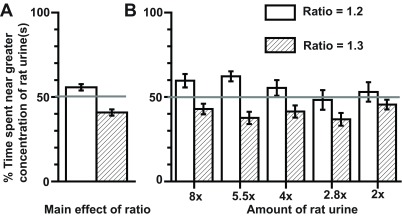
Discrimination threshold in mice is proportional to kairomone strength (mean±SEM). The discrimination threshold at varying doses of rat urine was further examined by setting up an avoidance-avoidance conflict, where mice chose to spend time in arms containing either lower or higher amounts of rat urine. The higher dose was of either a 20% (un-shaded bars) or 30% (shaded bars) greater magnitude (e.g. 120% or 130% of 2X). Abscissa depicts the lower dose used in each of the comparisons (e.g. 2X). Ordinate depicts time spent in arm with the greater amount of rat urine divided by the sum of the time spent in both arms (gray line = 50% chance). N = 15 mice for all comparisons.Log _2_ scale; arbitrarily set as 3.125 µl of rat urine.

The discrimination threshold (i.e the ‘just-noticeable difference’) was studied for five equidistant doses (Log _2_ scale) encompassing the linear part of the dose-response curve ( Figure 2). One arm of the arena in this case contained the dose depicted in the abscissa. The discrimination around this dose was studied in two successive trials by providing a greater amount of kairomone in the opposing arm, differing by a ratio of either 1.2 or 1.3. A positive discrimination was noted as less time was spent in the arm containing the greater volume of urine.

A two-way ANOVA for dose and ratio revealed a significant main effect of the ratio ( Figure 2A; F _(1,138)_ = 32.1, *p* = 0.00000008). The main effect of doses themselves did not reach statistical significance (F _(4,138)_ = 1.347, *p* = 0.256). Similarly, interaction between doses and ratios was not significant (F _(4,138)_ = 1.214, *p* = 0.308). Thus, regardless of the dose studied, discrimination threshold was constantly proportional to the kairomone strength ( Figure 2B) by a ratio ≤ 1.3 but above >1.2. In other words, the discrimination threshold was smaller for weaker stimuli and bigger for stronger stimuli.

## Discussion

Kairomones are compounds emitted by one species and co-opted by another receiving species, resulting in benefit for the receiver and detriment for the emitter. Kairomones can be used by both predators to locate prey and by prey to secure advanced warning of predator presence. Odors used as pheromones in intra-species communication are often the most vulnerable for co-option as kairomones. This is because use in conspecific communication requires robust expression of odors, making them more liable for eavesdropping by other species (reviewed in Kolluru and Zuk ^20^). In agreement with this formulation, rats use urine marks to communicate status and sexual attractiveness ^21– 23^; and, proteins secreted with rat urine are sufficient to initiate innate avoidance in mice ^13^, a prey species of rats ^15– 17^. The salient kairomone in this case has been identified to be a major urinary protein, MUP13 ^13^. These involatile rodent kairomones provide a unique opportunity to study kairomonal perception by virtue of their stability in the ambient environment and due to their ease of reliably controlling their dosing. This is in contrast with the volatile nature of many kairomones that require onerous delivery methods using olfactometers.

Several pheromonal responses in insects and mammals show dose responsivity whereby a stronger pheromonal stimulus evokes a greater response (e.g. Coureaud *et al.*, He *et al.* and Perna *et al.* ^24– 26^). In contrast, the dose responsivity of kairomonal communication has not been well-studied. In this report, we demonstrate that mice perceive rat kairomones in a dose-dependent manner. We speculate that the relative nature of kairomonal communication permits prey to calibrate foraging responses according to perceived predatory threats. For example, a weak kairomonal stimulus might signal the passage of a long period of time since the urine mark was laid, and thereby evoke lesser avoidance. In contrast, a stronger odor is likely to be fresh and a better indicator of predator presence. Similar dose-responsivity has been previously described during the perception of cat odors by rats ^14^.

The ability to calibrate avoidance also suggests that mice are able to differentiate between various amounts of kairomones. Animals can indeed discriminate between different amounts of many percepts, including olfactory sensations ^3– 7^. In the absence of an absolute numerical system, these discriminations are often dependant on a relative estimation based on comparative perceptions. Weber and Fletcher ^8^ formalized one of the hallmarks of such relative estimation by showing that the discrimination threshold is a constant fraction of the stimulus intensity in many perceptual systems ( *k* = ∆I/I; where k is a constant, I is intensity and ∆I is just-noticeable difference). In this report, we demonstrate that kairomonal communication in mice follows Weber’s law with Weber’s fraction valued at greater than 0.2 but smaller than 0.3. Weber’s law has been previously studied in human olfaction for volatile odors, yielding a comparable sensitivity of 0.28 ^7^. Similarly, pheromonal communication in Argentine Ants ( *Linepithema humile*) has been recently shown to follow Weber’s law ^26^. To the best of our knowledge, this is the first demonstration of Weber’s law applied to kairomones.

Mouse responsivity and discrimination threshold data to rat kairomones Dose responsiveness file: Dose responsiveness of mice (n=10) to differing volumes (ul) of rat urine (kairomone) or control (PBS). Time was measured in seconds; total time in the enclosure was 600 seconds. Mice were exposed to concentrations in ascending order, with a 24 hour gap between tests. Data corresponds to Fig 1 in the main manuscript.
Single dose responsiveness file: Responsiveness of mice (n=5) to a single volume (ul) of rat urine (kairomone) or control (PBS) over 5 days. Time was measured in seconds; total time in the enclosure was 600 seconds. Mice were exposed to concentrations in ascending order, with a 24 hour gap between tests.
Detection threshold: Discrimination threshold of mice (n=15) when exposed to two different volumes (ul) of rat urine (kairomone): a specific volume vs. either a 20% or a 30% increase in this volume. Time was measured in seconds; total time in the enclosure was 600 seconds. Mice were exposed to concentrations in ascending order, with a 24 hour gap between tests. Data corresponds to Fig 2 in the main manuscript.
