# Supplementary material for: Kairomonal communication in mice is concentration-dependent with a proportional discrimination threshold
Source: F1000Res. 2013 Dec 9;2:195. Originally published 2013 Sep 23. [Version 2] doi: 10.12688/f1000research.2-195.v2 (PMC3892922; doi:10.12688/f1000research.2-195.v2)
Supplement: Peer review history

# Peer review: Referee response for version 2

**Published:** 2013-12-30

**Authors:** Kazumi Osada

**Affiliations:** 1 Division of Physiology, Health Sciences University of Hokkaido, Hokkaido, Japan

Although I do not feel the paper is perfectly acceptable, I admit it has been improved by the authors, and am not inclined to reject the paper anymore.

I have read this submission. I believe that I have an appropriate level of expertise to confirm that it is of an acceptable scientific standard.

# Peer review: Referee response for version 2

**Published:** 2013-12-10

**Authors:** Joanne Yew

**Affiliations:** 1 Temasek Life Sciences Laboratory, National University of Singapore, Singapore, Singapore

My concerns have been adequately addressed - thank you for doing so.

I have read this submission. I believe that I have an appropriate level of expertise to confirm that it is of an acceptable scientific standard.

# Peer review: Referee response for version 2

**Published:** 2013-12-10

**Authors:** Fernando Martínez-García, Lluis Fortes-Marco

**Affiliations:** 1 Faculty of Biology, University of Valencia, Valencia, Spain

After the comments and amendments of the authors, it becomes now clear that they are testing the aversion of mice to different concentrations (rather than different volumes) of the high molecular weight fraction of rat urine, and they are doing so properly. 

Even if their results differ from ours, the quality of the paper is high and the results are of interest for the researchers in the area of communication through chemosignals in the rodent model.

We wish the authors good luck in their future research.

We have read this submission. We believe that we have an appropriate level of expertise to confirm that it is of an acceptable scientific standard.

# Peer review: Referee response for version 1

**Published:** 2013-11-12

**Authors:** Fernando Martínez-García, Lluis Fortes-Marco

**Affiliations:** 1 Faculty of Biology, University of Valencia, Valencia, Spain

The manuscript by Vasudevan and Vyas explores the interesting hypothesis that the response of mice to urine-borne kairomones of rats is dose dependent according to the Weber’s law. To do so the authors basically analyse the avoidance of mice to different amounts of urine. In fact, the authors purify the MUPs fraction (>3 kD mw), where the kairomone is contained according to Papes *et al.* (2010), and they treat this fraction with high concentrations of menadione to displace odorants contained in the lipophilic pockets of the MUPs. This strategy ensures that the reaction of mice to the stimulus is due to the major urinary proteins and not to urine-borne volatiles.

The results apparently lend support to the authors’ hypothesis. There is a highly significant (negative) correlation between the amount of stimulus and the time spent near it by the mice, following a sigmoid dose-response curve.

The authors further test their hypothesis by checking whether mice are able to discriminate different amounts of rat kairomone. To do so they design a simple two-choice test in which the mice can explore two arms of an arena in which two different amounts of MUPs are present, the ratio between the amount in both sides being 1.2 or 1.3. There is a clear, highly significant avoidance of the arm containing the higher amount, as compared to the other arm. This demonstrates that, throughout the linear region of the dose-response curve, mice discriminate two “doses” differing by x1.2 or x1.3.

The main problem with this experimental design is that the authors are checking the response to different amounts (from 3.125 to 50 μL) of the putative kairomone, rather than to different concentrations of it. If the kairomone were volatile, there would be a direct relationship between amount of sample (volume) and the concentration of the substance in the air: the larger air-liquid interface, the more molecules would be in the air phase. However, a MUP is, due to its high molecular weight, virtually non-volatile. Being a vomeronasal stimulus (according to Papes *et al.,* 2010, trp2 ^-/-^ do not avoid rat urine), for the animal to detect the kairomone it must contact the “drop” of MUP solution and perform vomeronasal pumping.

The important question here is how the MUP solution is presented, and no details are found in Material and Methods. Very likely, a drop of the MUP solution would be deposited in a piece of filter paper or a similar absorbent material. Depending on the volume of solution, a given area of the paper would become soaked by capillarity.

In a regular piece of absorbent material, a drop of liquid would diffuse giving an approximately circular wet area. Accordingly, one can calculate the theoretical relationship between the volume (V) of MUP solution and the radius (r) of the resulting spot (circular wet area) which would be related to the function: V = π·r ^2^. The radius of the spot of urine increases with the volume of the drop. Depending on the size of the area considered in each experiment as “near urine”, this fact, rather than the effect of the “dose”, may explain the results.

In Material and methods, under the heading “Dose-responsivity to kairomones”, the authors explain that “avoidance was quantified by comparing the time spent by mice in two opposing bisects of the area (76x9 cm; 15 cm)”. The term bisect is confusing (it may mean a line sectioning something in two halves, or just sectioning it in two pieces of different size). It is difficult, therefore, to have a clear idea of what the authors are really measuring, for two reasons. First, in spite of what they say (“…comparing the time spent by mice in two opposing bisects”) in their Fig. 1 the authors only represent the time spent “near rat urine”. It seems that this is the measurement analysed statistically (no comparison is made with the side containing PBS).

Second, the arena employed for the tests seems to be a long corridor 76cm long per 9 cm wide, but there is no mention of the actual size of the area that the authors consider “near rat urine”. I assume that they are using an identical area throughout the experiment, maybe a given region at the end of the arm where urine (or MUP solution) is presented. But, if so, as the volume of urine (or MUP solution) increases, the urine spot occupies a larger proportion of the area “near rat urine”. If we assume that contacting the urine allows detecting the kairomone (by vomeronasal pumping) and results in avoidance, the larger the urine spot (which would be the region actually avoided), the shorter the time in the area “near rat urine”. When the spot diameter approaches the size of the area “near rat urine” (e.g. the width of the arena), the time visiting this area reaches an asymptotic line at a minimum. This would explain Figure 1 of the manuscript.

In the same vein, if two spots of different size are located in the two sides of the arena (experiment 2; discrimination threshold) and animals are avoiding contact with the MUP spot, the time in area “near rat urine” on the side having a larger spot (let’s say a 70% of the area “near rat urine”) will be shorter than the time in the side having a smaller spot (which may represent just a 20% of the area “near rat urine”).

As a conclusion, the main problem of the work reported in the manuscript, otherwise well designed and performed, is that the authors are using the term “dose” in a misleading way. The volume of MUP solution is probably not a proper measurement of the “dose” of the stimulus, or the response of the sensory organ (VNO). Vomeronasal neurons respond to the stimulus (MUP) in a concentration-dependent manner (e.g. Leinders-Zufall *et al.,* 2000; Leinders-Zufall *et al.,* 2004), rather than in a way proportional to the volume of the MUP solution (and the area of the resulting “urine” spot). Therefore, varying the concentration of MUPs in the solution while using a fixed volume would really test the hypothesis that the dose-response to a kairomone follows Weber’s law.

Anyway, the results are difficult to interpret due to the lack of details on procedure. The way in which the stimulus is presented is not properly described, and the size of the area “near rat urine” must be known in order to check if the explanation of the results by the authors is correct or not.

We have read this submission. We believe that we have an appropriate level of expertise to confirm that it is of an acceptable scientific standard, however we have significant reservations, as outlined above.

# Response

**Published:** 2013-11-30

**Authors:** Ajai Vyas

**Affiliations:** Nanyang Technological University, Singapore

We have attempted to address the concerns of referee #3 in second version of the manuscript. The referee’s comments are based on two strands of criticisms, both of which arise because of our inadequate and unclear description of methods:

- The assumption that the authors varied volume in order to vary amount of the stimulus, rather than concentration.
- The bisect area was undefined in the original methods.

The volume of urine stimuli used was constant; concentrations were varied instead to reach varying amounts. This distinction has now been made clearly in the revised version. Since the volume of the spot was constant, the radius/area of the spot of urine remains constant across the different doses, which addresses the question of variance in the area of the spot of urine affecting our results.

In addition, we have defined the time spent near rat urine by providing dimensions of bisects. In this case, bisects were obtained by equal division of the arena. This point has been clearly mentioned now in the revision. A rather big difference in size of bisect and size of the stimulus spot, along with equal volume of stimuli across doses, avoids substantial interaction between doses and bisect definition.

We believe additional clarifications and elaborations in the material and methods sections adequately respond to all of referee’s comments.

In figure 1, we did not show data for PBS because time spent near PBS is directly dependent on time spent neat rat urine stimuli. We use two equal bisects to measure time spent near PBS and rat odor (i.e. the arena is virtually divided in two equal compartments). In this design, time spent near PBS is merely subtraction of time near rat odor from total trial duration. Dependence of these two measures then means that statistics for both of them will be redundant.

# Peer review: Referee response for version 1

**Published:** 2013-10-25

**Authors:** Kazumi Osada

**Affiliations:** 1 Division of Physiology, Health Sciences University of Hokkaido, Hokkaido, Japan

This paper showed the quantitative relationship between the amount of crude rat urinary kairomone fraction, and aversive behavior in mice. The figure 1 data is reasonable, but not interesting.

The figure 2 data fails to demonstrate the relationship between the amount of rat urine and the "detection threshold" of the mice. On top of that, this evaluation system is not suitable for detecting the detection threshold of kairomones. This is because if mice spent time evenly in this system, you cannot say that the mice cannot distinguish between these two sources, you can only say that the mice did not distinguish between them.

I have read this submission. I believe that I have an appropriate level of expertise to state that I do not consider it to be of an acceptable scientific standard, for reasons outlined above.

# Response

**Published:** 2013-11-30

**Authors:** Ajai Vyas

**Affiliations:** Nanyang Technological University, Singapore

We believe revisions to the script address the main concerns raised by referee #2.

The referee posits that creating an avoidance-avoidance conflict in figure two does not differentiate between possibilities that mice cannot detect rat odor, versus that mice avoid both sources of odor with equal magnitude. Our goal in this figure is not the detection but the discrimination between sets of two odor source. The detection itself is being tested in Figure 1, which does not use avoidance-avoidance conflict.

 In other words, we are asking if mice can discriminate an odor (i.e. baseline) from another odor that is incrementally stronger and is presented simultaneously (baseline X ∆). Weber’s law predicts that increment in odor strength required for discrimination will be proportional to baseline (i.e. ∆ remains constant). Because Weber’s law pertains to discrimination and not detection, we tested our hypothesis in Figure 2 using avoidance-avoidance conflict.

We have revised the manuscript to incorporate “discrimination” instead of “detection” in the appropriate results and figure sections.

# Peer review: Referee response for version 1

**Published:** 2013-10-14

**Authors:** Joanne Yew

**Affiliations:** 1 Temasek Life Sciences Laboratory, National University of Singapore, Singapore, Singapore

This article addresses and characterizes rat urine kairomonal effects on mouse avoidance behavior. The study convincingly shows that 1) the effect is dose-dependent and 2) mice display proportional discrimination across several different concentrations.

The title of the study and abstract provide an adequate summary. With respect to the methods, I would like some clarification on whether the different urine doses were made from the same initial sample. If several different urine collections were used, it should be stated how it was verified that the concentration of MUPs in the size exclusion fraction is similar from sample to sample.

Otherwise, the conclusions are justified on the basis of the results and the paper is well-written.

I have read this submission. I believe that I have an appropriate level of expertise to confirm that it is of an acceptable scientific standard.

# Response

**Published:** 2013-11-30

**Authors:** Ajai Vyas

**Affiliations:** Nanyang Technological University, Singapore

The same urine sample was indeed used to create all doses during this experiment. The second version of the manuscript now clearly states this in material and methods section.
